# MicroRNA-135b, a HSF1 target, promotes tumor invasion and metastasis by regulating RECK and EVI5 in hepatocellular carcinoma

**DOI:** 10.18632/oncotarget.2965

**Published:** 2014-12-11

**Authors:** Yan Li, Dan Xu, Chunyang Bao, Yuannv Zhang, Di Chen, Fangyu Zhao, Jie Ding, Linhui Liang, Qifeng Wang, Li Liu, Jinjun Li, Ming Yao, Shenglin Huang, Xianghuo He

**Affiliations:** ^1^ State Key Laboratory of Oncogenes and Related Genes, Shanghai Cancer Institute, Renji Hospital, Shanghai Jiao Tong University School of Medicine, Shanghai, China; ^2^ Fudan University Shanghai Cancer Center and Institutes of Biomedical Sciences, Department of Oncology, Shanghai Medical College, Fudan University, Shanghai, China

**Keywords:** MicroRNA-135b, RECK, EVI5, HSF1, Hepatocellular Carcinoma

## Abstract

MicroRNAs (miRNAs) often localize to chromosomal fragile sites and are associated with cancer. In this study, we screened for the aberrant and functional miRNAs in the regions of copy number alterations (CNAs) in hepatocellular carcinoma (HCC), and found that miR-135b was frequently amplified and upregulated in HCC tissues. The expression level of miR-135b was inversely correlated with the occurrence of tumor capsules. In addition, miR-135b promoted HCC cell migration and invasion *in vitro* and metastasis *in vivo*. The reversion-inducing-cysteine-rich protein with kazal motifs (RECK) and ecotropic viral integration site 5 (EVI5) were identified as the direct and functional targets of miR-135b in HCC. Furthermore, we observed that heat shock transcription factor 1 (HSF1) directly activated miR-135b expression, consequently enhancing HCC cell motility and invasiveness. The newly identified HSF1/miR-135b/RECK&;EVI5 axis provides novel insight into the mechanisms of HCC metastasis, which may facilitate the development of new therapeutics against HCC.

## INTRODUCTION

Hepatocellular carcinoma (HCC) accounts for 85–90% of primary liver cancers and is the third most common cause of cancer mortality[1]. HCC occurs within an established background of chronic liver disease and cirrhosis, with major risk factors being hepatitis B and C viruses, alcohol, aflatoxin B, and non-alcoholic fatty liver disease. However, the molecular pathogenesis of HCC is still largely unknown. Genomic instability is a hallmark of cancers, and recurrent chromosomal alterations often occur in tumors[2]. We previously identified 1241 recurrent regions of somatic copy number alterations (CNAs) in HCC primary tumors using a whole-genome Affymetrix SNP 6.0 array[3]. Several functional protein-coding genes, *TRIM35, HEY1, SNRPE, MPZL1* and *SERPINA5* were identified in these somatic CNAs[3-5]. Nevertheless, it has also been reported that many miRNAs in genomic fragile sites play vital roles in human cancer[6].

MicroRNAs (miRNAs) are small, endogenous, non-coding RNAs that play important regulatory roles in organisms by targeting mRNAs for cleavage or translational repression[7]. Bioinformatic analyses suggest that more than 60% of protein-coding genes may be directly targeted by miRNAs[8]. Meanwhile, a single mRNA can be modulated by multiple miRNAs. Growing evidence has demonstrated that these small RNAs are involved in almost every aspect of biological processes and human diseases, including cancer[9, 10]. In cancer, miRNAs can function as oncogenes or tumor suppressors[11]. Recent studies have shown that many miRNAs localize to chromosomal fragile sites or breakpoints[6], indicating that they may be important in cancer development and progression. For example, the tumor suppressors miR-15a and miR-16-1, located at 13q14.3, are frequently deleted and downregulated in chronic lymphocytic leukemia[12], while the gain of miR-151 and its host gene PTK2 on chromosome 8q24.3 facilitates HCC cell migration and metastasis[13]. However, the identity of the aberrant miRNAs in HCC CNVs remains to be comprehensively elucidated.

In this study, aberrantly expressed miRNAs located in the 1241 CNAs of HCC were screened. MiR-135b, which was frequently amplified and upregulated in HCC tissues, promoted HCC invasion and metastasis through regulation of reversion-inducing-cysteine-rich protein with kazal motifs (RECK) and ecotropic viral integration site 5 (EVI5) expression. Moreover, the transcription factor heat shock transcription factor 1 (HSF1) directly activated miR-135b expression and subsequently promoted HCC cell motility and invasiveness, revealing a novel mechanism for HSF1-mediated cancer progression that involves regulation by miRNA.

## RESULTS

### MiR-135b is upregulated in HCC and its expression is inversely correlated with tumor capsule occurrence

We previously identified 1241 regions with somatic CNAs in HCC[3]. A search of the UCSC (GRCh37/hg19) database revealed that about 58% (720/1240) of miRNAs were among the 1241 CNAs (P <0.001, χ^2^ test), suggesting that miRNAs frequently localize to chromosomal fragile sites in HCC. Of the miRNAs, 92 were present in recurrent (≥ 30%) CNAs. After screening expression levels and excluding reported miRNAs, 18 were found to have CNAs, and their relationship to miRNA transcript expression was examined (Supplementary Figures S1 and 2). A concordance was found between mature miRNA expression and genomic DNA levels for 44% (8/18) of the miRNA genes (Supplementary Table S1). We further performed functional assays to determine the potential role of these miRNAs in HCC cells. Transwell assays revealed that miR-92b, -135b, -875, and -548a enhanced and miR-320b suppressed cell migration (Supplementary Figure S3 and Supplementary Table S1). In addition, the cell proliferation assay showed that miR-92b promoted, while miR-765, -205, and miR-548a inhibited cell growth (Supplementary Figure S4 and Supplementary Table S1).

**Figure 1:**
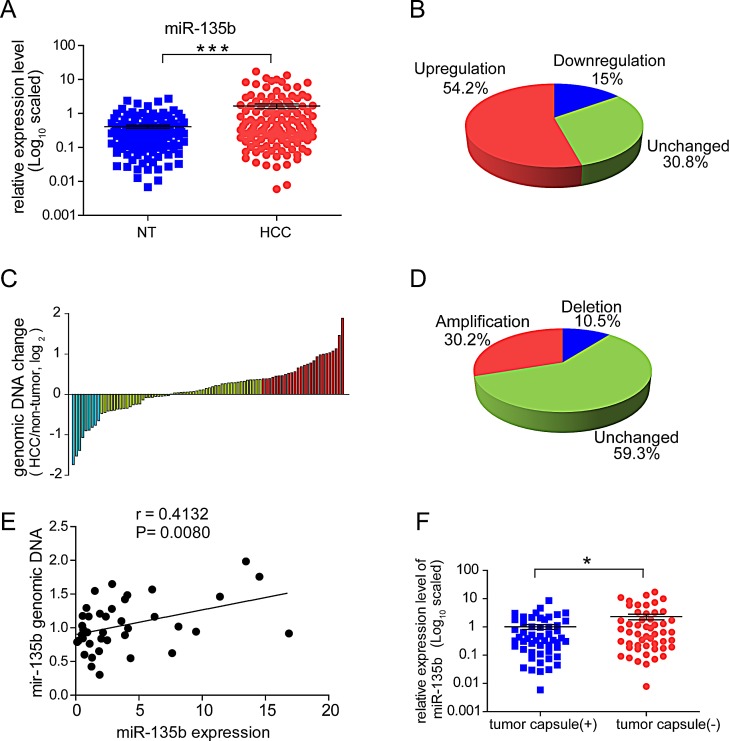
MiR-135b is upregulated in hepatocellular carcinoma (HCC) and its expression is inversely correlated with tumor capsule occurrence (A) MiR-135b expression was assessed by TaqMan real-time PCR relative to the internal standard small nuclear U6B RNA (RNU6B) in 120 paired HCC and adjacent non-cancerous liver tissue (NT) specimens. A significant upregulation of miR-135b expression in paired samples was defined as fold change > 2. P < 0.05 (paired *t*-test) was considered statistically significant. (B) Pie chart of the proportions of HCC samples in which miR-135b expression was upregulated (red), downregulated (blue), or showed no change (green). (C) Real-time PCR analysis of miR-135b DNA copy numbers in 86 paired HCC and matched non-tumor tissues normalized to β-actin. Data are presented as the log_2_ fold change. (D) Pie chart of the proportions of chromosomal regions that are frequently amplified (red), unchanged (green), and deleted (blue) in HCC samples. (E) Correlation between miR-135b expression level (x) and genomic DNA content (y) in HCC tissue samples (n = 36), with RNU6B and β-actin serving as an internal controls, respectively. Statistical analysis was performed with Pearson's correlation analysis. (F) Expression level of miR-135b relative to RNU6B in HCC patient samples with (n = 53) or without (n = 52) tumor capsules. P < 0.05 (Student's *t* test) was considered statistically significant.

MiR-135b was the most frequently upregulated miRNA among those examined (57%; Supplementary Table S1). In another independent cohort of HCC samples, miR-135b expression was observed markedly upregulated in primary HCC compared to noncancerous tissue (Figure 1A). When comparing primary cancers with their corresponding non-tumorous tissues, the upregulation of miR-135b (greater than a two-fold change) was observed in 54.2% (65/120) of HCCs (Figure 1B). An analysis of the genomic region revealed that the miR-135b locus is amplified in approximately 30.2% (26/120) of HCC tissues (Figures 1C and D). The up-regulated miR-135b expression was correlated with its genomic content in HCCs (Figure 1E), suggesting that increased expression of miR-135b may result from the amplification of its corresponding chromosomal region. Furthermore, we analyzed the correlation between the expression level of miR-135b and clinic pathological features of HCC (Supplementary Table S2). Upregulation of miR-135b level was found to be inversely correlated with tumor capsule occurrence (P=0.04) and serum hepatitis B virus E antigen level (P=0.036). MiR-135b expression was significantly upregulated in HCC patient samples without tumor capsule (P=0.0212) (Figure 1F), suggesting that miR-135b may play a role in tumor invasion.

### MiR-135b promotes HCC cell migration and invasion *in vitro* and metastasis *in vivo*

**Figure 2:**
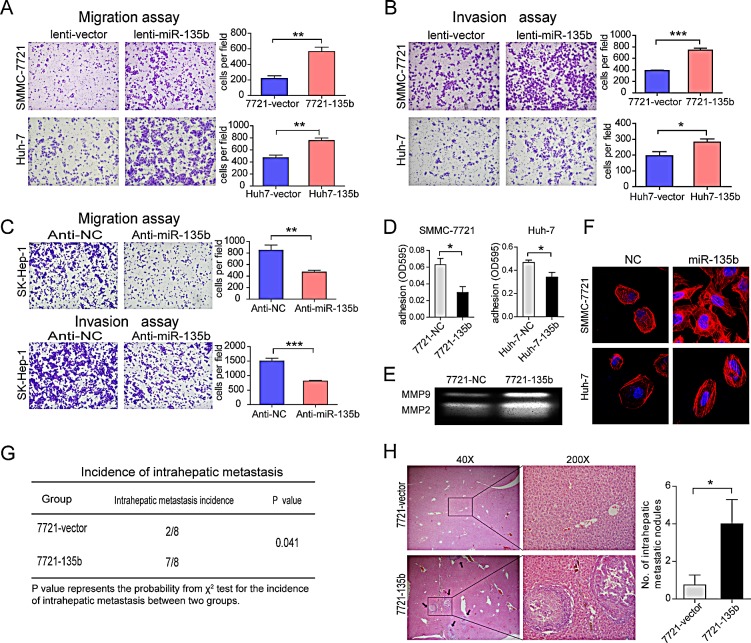
MiR-135b promotes hepatocellular carcinoma (HCC) cell migration and invasion *in vitro* and metastasis in vivo (A–C) Transwell migration and invasion assays of SMMC-7721 (A) and Huh-7 (B) cells after lentiviral transduction of miR-135b or empty vector, and in SK-Hep-1 cells (C) after transfection with a miR-135b inhibitor or negative control. Results were pooled from at least three independent experiments; statistical analysis was performed with Student's *t* test. *P < 0.05; **P < 0.01; ***P < 0.001. Data represent the mean ± SEM. (D) Adhesion assay of SMMC-7721 and Huh-7 cells after transfection with miR-135b mimic or negative control (NC). MiR-135b could significantly decrease the cell-matrix adhesion. Data represent the mean ± SEM. *P < 0.05(Student's *t* test). (E) Matrix metalloproteinase MMP2 and MMP9 activities were assessed by gelatin zymography in SMMC-7721 cells after transfection with miR-135b mimic or NC. (F) SMMC-7721 and Huh-7 cells were treated with phalloidin and 4′6-diamidino-2-phenylindole to label F-actin (red) and nuclei (blue), respectively, and visualized by confocal microscopy. (G) Effects of miR-135b overexpression on the in vivo metastatic potential of SMMC-7721 cells in xenograft mouse models (n = 8 per group). P < 0.05 was considered statistically significant (χ^2^ test). (H) Hematoxylin and eosin-stained sections of liver tissue from mice 8 weeks after transplantation of SMMC-7721-vector or SMMC-7721-135b cells. The black arrow indicates a tumor formed in the liver. Intrahepatic nodules were quantified and analyzed using Student's *t* test. *P < 0.05.

To better evaluate the biological functions of miR-135b in HCC, we first detected the expression of miR-135b in various HCC cell lines. The results showed that miR-135b expression level was relatively low in SMMC-7721 and Huh-7 cells, whereas it was relatively high in SK-HEP-1 cells (Supplementary Figure S5A). We therefore constructed a lentivirus vector harboring miR-135b and established two stable cell lines after lentivirus transduction in SMMC-7721 and Huh-7 cells (Supplementary Figure 5B). Transwell assays demonstrated that miR-135b could promote cell migration and invasion in both SMMC-7721 and Huh-7 cells (Figures 2A and B). In contrast, the silencing of endogenous miR-135b in SK-HEP-1 cells suppressed cell migration and invasion (Figure 2C). We noted that miR-135b had no significant effect on the proliferation of HCC cells (Supplementary Figures S6A and B). Thus, the stimulatory effects of miR-135b on cell migration and invasion were not caused due to an increased in cell numbers. We observed that miR-135b inhibited cell-matrix adhesion in SMMC-7721 and Huh-7 cells (Figure 2D). Gelatin zymograph assay showed that miR-135b enhanced the activities of MMP9 and MMP2 (Figure 2E). Moreover, the formation of stress fibers was increased in cells expressing miR-135b by immunofluorescence (Figure 2F). These findings indicated that miR-135b could influence cell adhesion, MMP secretion and cell morphology to facilitate cell migration and invasion.

To evaluate the role of miR-135b in tumor invasion and metastasis *in vivo*, SMMC-7721 cells stably expressing miR-135b or control cells were transplanted into the livers of nude mice, which were examined 8 weeks later. Metastatic nodules were observed in the liver of a higher proportion of mice in the miR-135b than in the control group (87.5% vs. 25%; P= 0.041) (Figures 2G and H). Taken together, these findings indicate that miR-135b promotes cell migration and invasion *in vitro* and metastasis *in vivo*.

### RECK and EVI5 are direct, downstream targets of miR-135b in HCC

**Figure 3:**
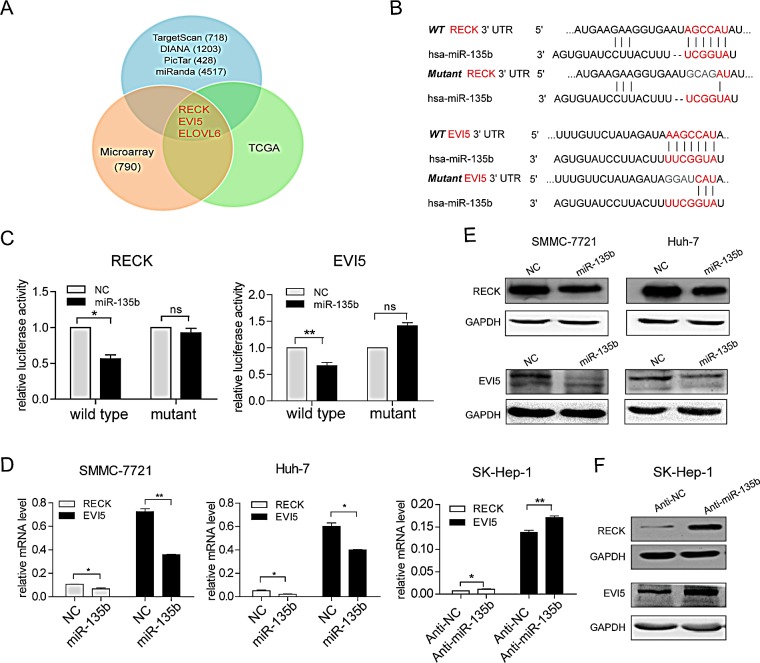
RECK and EVI5 are direct downstream targets of miR-135b in hepatocellular carcinoma (HCC) (A) Candidate genes identified by prediction algorithms, gene expression profiles and transcript expression level in The Cancer Genome Atlas (TCGA; http://tcga.cancer.gov). (B) Putative miR-135b binding sites in the RECK and EVI5 3′ untranslated region (UTR). Mutant binding sequences were highlighted in gray. (C) Firefly luciferase activity normalized to Renilla luciferase activity in HEK-293T cells co-transfected with luciferase reporters with wild-type or mutant 3′ UTR of RECK or EVI5 along with miR-135b mimic or negative control (NC). *P < 0.05; **P < 0.01; ***P < 0.001. Data represent the mean ± SEM. (D) RECK and EVI5 transcript expression level were assessed by real-time PCR after transfection with miR-135b mimic or NC in SMMC-7721 and Huh-7 cells, and in SK-Hep-1 cells after transfection with miR-135b inhibitor or negative control. *P < 0.05; **P < 0.01; ***P < 0.001. Data represent the mean ± SEM. (E,F) RECK and EVI5 protein levels were determined by western blotting following transfection of SMMC-7721 and Huh-7 cells with miR-135b mimic or NC (E) or transfection of SK-Hep-1 cells with a miR-135b inhibitor or NC (F). Glyceraldehyde-3-phosphate dehydrogenase (GAPDH) served as a loading control.

To investigate the mechanistic basis of the miR-135b-mediated increase in HCC cell motility, we employed three strategies to identify the potential downstream targets of miR-135b: first, performing database searches in miRNA target prediction programs; second, analyzing gene expression profile induced by miR-135b; third, focusing on the mRNAs that are suppressed by miR-135b and downregulated in HCC tissues (Figure 3A). Among them, three genes (RECK, EVI5 and ELOVL6) were found to be the candidates of miR-135b targets. We noted that there was a negative correlation between miR-135b expression and mRNA levels of RECK and EVI5 in HCC samples (Supplementary Figure S7C). Moreover, transwell assays showed that RECK and EVI5, but not ELOVL6, were involved in the suppression of HCC cell migration and invasion (Supplementary Figure S7D). Thus, RECK and EVI5 were chosen as the potential functional targets of miR-135b in HCC. Both RECK and EVI5 mRNAs had one possible binding site for miR-135b (Figure 3B). To determine whether miR-135b directly regulated RECK and EVI5, the wild-type or mutated 3′ UTR of RECK and EVI5 mRNA were inserted downstream of the luciferase reporter gene, and miR-135b mimic was co-transfected with each construct in HEK-293T cells. MiR-135b reduced the luciferase activity of wild-type RECK and EVI5 3′ UTRs, but this decrease was less pronounced for UTRs with mutated binding sites (Figure 3C). Meanwhile, we synthesized a compensatory mutation in miR-135b, and found that the compensatory mutation in miR-135b could restore the repression of luciferase reporter with the target genes' 3′UTR (Supplementary Figures S8A and B). Endogenous RECK and EVI5 mRNA or protein levels were also markedly decreased in SMMC-7721 and Huh-7 cells following the transfection of miR-135b mimic (Figures 3D and E), while miR-135b inhibition increased the levels of these mRNAs and proteins in SK-HEP-1 cells (Figures 3D and F). These results demonstrate that miR-135b directly inhibits the expression of RECK and EVI5 via regulatory interactions with their 3′ UTRs.

### MiR-135b promotes HCC cell migration and invasion through inhibiting RECK and EVI5

**Figure 4:**
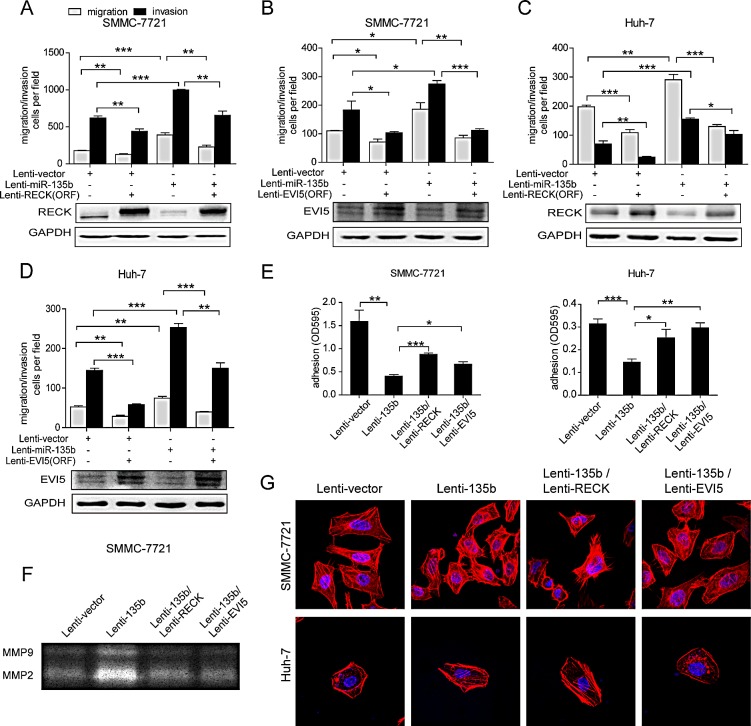
MiR-135b promotes HCC cell migration and invasion via regulation of RECK and EVI5 (A–D) Transwell migration and invasion assays of SMMC-7721-vector and SMMC-7721-135b (A, B) and Huh-7-vector and Huh-7-135b (C, D) cells following transduction with RECK and EVI5 open reading frames lacking the 3′ untranslated region or with a control lentivirus. *P < 0.05; **P < 0.01; ***P < 0.001 (Student's t test). Data represent the mean ± SEM. (E) Adhesion assay performed after restoring RECK and EVI5 expression in SMMC-7721 and Huh-7 cells overexpressing miR-135b. Data represent the mean ± SEM. *P < 0.05; **P < 0.01; ***P < 0.001 (Student's t test). (F) Matrix metalloproteinase (MMP)2 and 9 activities were assessed by gelatin zymography after restoration of RECK or EVI5 expression in SMMC-7721 and Huh-7 cells overexpressing miR-135b. (G) SMMC-7721 and Huh-7 cells overexpressing miR-135b were treated with phalloidin and 4′6-diamidino-2-phenylindole to label F-actin (red) and nuclei (blue) after restoration of RECK and EVI5, and visualized by confocal microscopy.

RECK is known to suppress migration and invasion of cancer cells[14], but the function of EVI5 is less clear[15]. For a better understanding of the role of RECK and EVI5 in miR-135b-mediated tumor invasion, we first examined the expression and function of RECK and EVI5 in HCC. We observed that the expression levels of both RECK and EVI5 were significantly downregulated in HCC tissues compared with their noncancerous counterparts (Supplementary Figures S7A and B). Silencing of RECK and EVI5 by siRNA increased the motility and invasiveness of SMMC-7721 and Huh-7 cells, which was similar to the phenotype induced by miR-135b (Supplementary Figure S7D). Conversely, the ectopic expression of RECK and EVI5 inhibited HCC cell migration and invasion (Figure 4A and Supplementary Figures S8C and D).

To investigate whether RECK and EVI5 are effectors of miR-135b-induced HCC cell migration and invasion, RECK or EVI5 lacking the 3′ UTR was transduced into SMMC-7721-135b and Huh-7-135b stable cell lines. Restoring RECK and EVI5 expression abrogated the increase in cell motility and invasiveness (Figures 4A, B, C and D), and changes in adhesion and MMP2 and 9 activity (Figures 4E and F), induced by miR-135b. However, only EVI5 and not RECK blocked miR-135b-induced stress fiber formation (Figure 4G). These results demonstrate that RECK and EVI5 are functional targets of miR-135b in HCC cells.

### MiR-135b is regulated by the transcription factor HSF1

**Figure 5:**
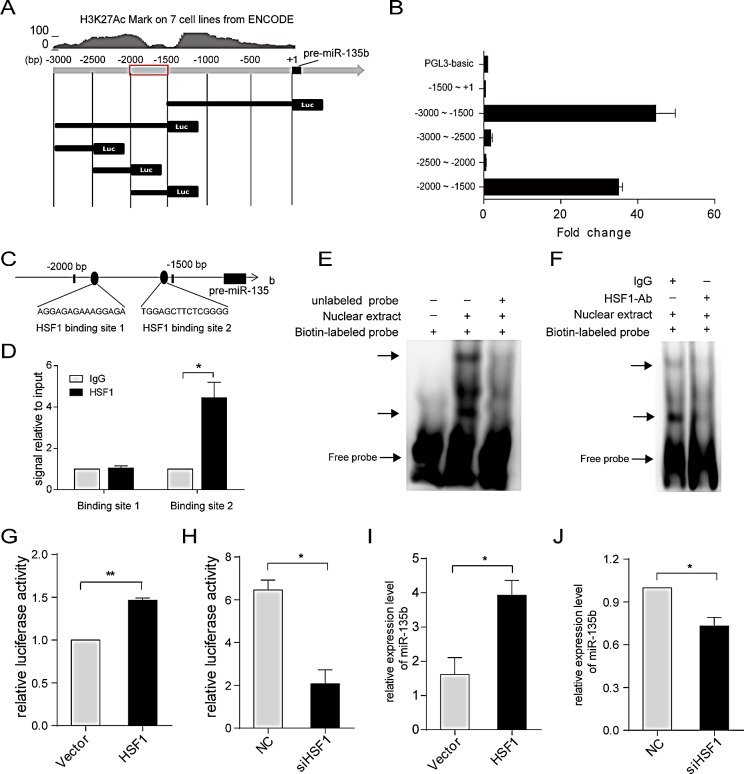
MiR-135b is regulated by heat shock transcription factor 1 (HSF1) (A) Schematic representation of human miR-135b promoter reporter constructs. Fragments of various lengths between −3000 to +1 bp of pre-miR-135b were cloned downstream of the firefly luciferase reporter. Upper panel shows the histone H3K27Ac mark detected in seven cell lines using the ENCODE genome browser. (B) Luciferase activity in SMMC-7721 cells transfected with firefly luciferase reporter plasmids containing various upstream regions of pre-miR-135b. Renilla luciferase reporter was cotransfected with pGL3-basic or plasmid reporter. (C) Putative HSF1 binding sites in the region between −2000 to −1500 bp upstream of pre-miR-135b. (D) Chromatin immunoprecipitation in SMMC-7721 cells, followed by real-time PCR amplification of two binding sites within the miR-135b promoter region. (E) EMSA was performed to verify the interaction of HSF1 binding site 2 with nuclear proteins which were prepared from SMMC-7721 cells. Incubations were performed in the presence (+) or absence (−) of 200-fold excess of unlabeled consensus oligonucleotide. DNA-protein complexes were fractionated by polyacrylamide gel electrophoresis and visualized by horseradish peroxidase-conjugated streptavidin. DNA–protein complexes were indicated by arrows. (F) Antibody-supershift assay demonstrated HSF1 was a potential nuclear protein interacting with predicted HSF1 binding site 2 sequences. The biotin-labeled intensity of the DNA–protein complexes decreased when HSF1 antibody was added. DNA–protein complexes were indicated by arrows. (G) Luciferase activity associated with the region between −2000 and −1500 bp of pre-miR-135b in SMMC-7721 cells transfected with HSF1. (H) Luciferase activity associated with the region between −2000 and −1500 bp of pre-miR-135b in SMMC-7721 cells transfected with small interfering RNA (siRNA) against HSF1 or with a negative control (NC). (I) MiR-135b expression in SMMC-7721 cells after *HSF1* overexpression as assessed by real-time PCR. (J) MiR-135b expression after *HSF1* was knockdown in SMMC-7721 cells. D, G-J, Data represent the mean ± SEM. *P < 0.05; **P < 0.01 (Student's t test).

MiR-135b expression is upregulated in 54.2% of HCC tissues, while only 30.2% of miR-135b genomic regions are amplified. Thus, additional mechanisms besides gene amplification are likely responsible for the dysregulation of miR-135b in HCC. An enrichment of the H3K27Ac histone mark, often found near active regulatory elements, was observed in the 3-kb region upstream of pre-miR-135b (Figure 5A). A series of luciferase reporter constructs containing fragments from this region (from −3000 to +1 bp) were cloned into the pGL3-basic vector (Figure 5A), and luciferase activity was measured after transfection of these constructs into SMMC-7721 cells. The highest activity was associated with the −2000 to −1500 bp fragment (Figure 5B), indicating that it contained regulatory elements critical for the transcription of miR-135b. Next, we used TFSEARCH[16] to predict the transcription factor binding sites in this region. We found this region contained two binding sites for HSF1 (Figure 5C). Chromatin immunoprecipitation (ChIP) assays revealed that HSF1 could bind to this region at binding site 2 in HCC cells (Figure 5D). We also found deletion of binding site 1 does not affect the reporter activities, but deletion of binding site 2 significantly reduces the activities (Supplementary Figures S9A and B). Moreover, both EMSA (Figure 5E) and antibody-supershift assays (Figure 5F) showed an interaction between HSF1 and the binding site 2 *in vitro*. When co-transfection of the luciferase reporter containing the binding site 2 with HSF1 expressing vector or siRNA against HSF1, we found HSF1 overexpression increased the reporter activities (Figure 5G), and inhibition of HSF1 decreased its activities (Figure 5H). Consistent with this result, we observed the level of miR-135b expression was significantly upregulated by ectopic expression of HSF1 (Figure 5I), and downregulated by silencing of HSF1 expression (Figure 5J). Taken together, these findings demonstrate that HSF1 directly regulates miR-135b expression by binding to its upstream region.

### HSF1 promotes HCC cell migration and invasion via miR-135b

**Figure 6:**
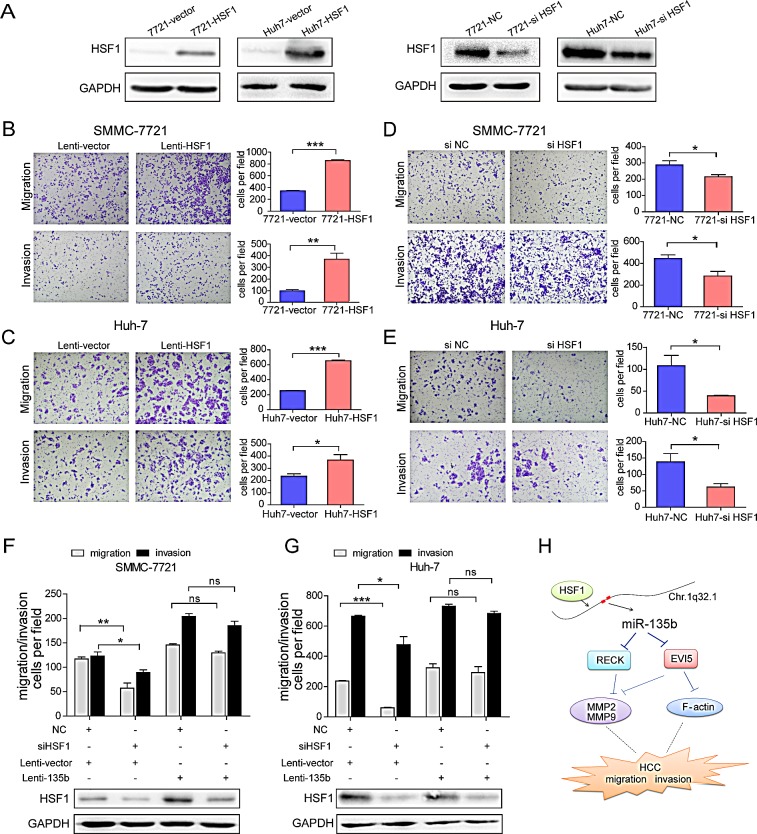
Heat shock transcription factor 1 (HSF1) promotes hepatocellular carcinoma (HCC) cell migration and invasion (A) HSF1 protein levels were determined by western blotting following lentiviral transduction of SMMC-7721 and Huh-7 cells with HSF1 or empty vector, and transfected with small interfering (si)RNA against HSF1 or a negative control (NC). (B–G) Transwell migration and invasion assays of SMMC-7721 and Huh-7 cells transduced with HSF1 or control lentivirus (B, C), and in SMMC-7721 and Huh-7 cells transfected with small interfering (si)RNA against HSF1 or a negative control (NC) (D, E), or after restoration of miR-135b following HSF1 knockdown (F, G). *P < 0.05; **P < 0.01; ***P < 0.001. Data represent the mean ± SEM. Statistical analysis was performed with Student's t test. (H) Model of miR-135b regulation and function in HCC.

HSF1 is a major transactivator of the cellular stress response and has been implicated in carcinogenesis in various organs[17]. To assess the function of HSF1 in HCC, SMMC-7721 and Huh-7 stable cell lines were established that overexpress HSF1 (Figure 6A). Cell migration and invasion were enhanced relative to control cells by HSF1 overexpression (Figures 6B and C); conversely, siRNA knockdown of HSF1 decreased cell motility and invasiveness in both SMMC-7721 and Huh-7 cells (Figures 6D and E). Furthermore, overexpression of miR-135b reversed the inhibitory effects of HSF1 knockdown on cell migration and invasion in HCC cells (Figures 6F and G; Supplementary Figures S10A and B). We also observed that the cell motility and invasiveness were reduced after miR-135b was inhibited in SMMC-7721 overexpressing HSF1 (Supplementary Figure S10C). These results indicate that HSF1-induced cancer progression via increased cell motility and invasion is mediated by miR-135b.

## DISCUSSION

In the present study, miR-135b was found through a screen of aberrantly expressed miRNAs in regions with CNAs to be frequently amplified and upregulated in HCC tissues. MiR-135b is upregulated in other types of cancers, including colorectal cancer (CRC)[18], non-small cell lung cancer[19], anaplastic larger cell lymphoma[20], and head and neck squamous cell carcinoma[21]. MiR-135b has been identified as a key downstream effector in oncogenic pathways and is a potential therapeutic target in the treatment of CRC[22]. However, a detailed understanding of miR-135b function and regulation in HCC is still lacking. MiR-135b is located at 1q32.1, and copy number gain of 1q is frequently observed in primary HCC[23]. The present results revealed that about two-thirds of HCC tissues had a high level of miR-135b expression (Figure 1). We found up-regulated miR-135b expression was correlated with its genomic content (Figure 1E). We also noted that HSF1 level was positively correlated with the expression of miR-135b in the HCC sample without miR-135b DNA amplification (Supplementary Figure S11). These findings indicated that miR-135b overexpression can partly be explained by genomic DNA alterations and *HSF1* activation. MiR-135b overexpression also enhanced the invasive and migratory potential of HCC cells without affecting proliferation (Figure 4), consistent with observations in other types of tumor with the exception of CRC cells, in which inhibiting miR-135b reduced tumor growth through regulation of genes involved in proliferation, invasion, and apoptosis[22].

RECK and EVI5 were identified as two targets of miR-135b in HCC cells (Figure 3). RECK is a cysteine-rich, extracellular protein with a protease inhibitor-like domain that exerts a tumor suppressor function[24] through negative regulation of MMPs and suppression of tumor invasion[25]. RECK is frequently downregulated in tumors[26-30], possibly as a result of transcriptional and post-transcriptional dysregulation[14, 31, 32]. This study identified miR-135b as a novel regulator of RECK. EVI5 belongs to a small subfamily of the Tre-2/Bub2/Cdc16 (TBC) domain-containing proteins that have diverse roles[15]. Early work associated with EVI5′s identification points towards its role as an oncogene or tumor suppressor[33, 34]. However, direct evidence for EVI5′s role in oncogenesis, or suppression of tumorigenesis, is still lacking[15]. Here we first reported EVI5 was frequently downregulated in HCC and functioned as a candidate metastasis suppressor for HCC. We have shown that miR-135b could bind directly to the 3′UTR of EVI5 and decrease the expression of EVI5 at both the mRNA and protein levels, thus providing the first line of evidence of EVI5 regulation at the post-transcriptional level by miRNA.

HSF1 is a highly conserved transcription factor that coordinates stress-induced transcriptional regulation and directs various physiological processes in eukaryotes[35]. HSF1 has also been implicated in malignant transformation, cancer cell survival, and tumor metastasis[36], but the precise mechanisms by which HSF1 promotes cancer progression remain poorly understood[35]. The central position of HSF1 in cellular homeostasis was well demonstrated, mainly through its strong effect in transactivating genes that encode heat shock proteins (HSPs)[17]; however, genome-wide surveys have revealed that HSF1 is extensively involved in transcriptional reprogramming of cancer-specific genes in tumorigenesis[37]. It was shown here that HSF1 directly activates miR-135b expression and thereby promotes HCC cell motility and invasiveness, demonstrating that miR-135b is a mediator of HSF1-induced cancer progression.

In conclusion, these findings indicate that the upregulation of miR-135b expression in HCC may result from genomic DNA amplification and HSF1 activation. MiR-135b promotes HCC cell migration and invasion *in vitro* and metastasis *in vivo* through direct regulation of its targets RECK and EVI5. This novel HSF1/miR-135b/RECK/EVI5 axis provides greater insight into the mechanism of HCC metastasis and can facilitate the development of new therapeutics to treat HCC.

## METHODS

### Cell culture

HEK-293T, Huh-7, HepG2, SK-HEP-1, PLC/PRF/5, MHCC-LM3, SMMC-7721, Hep3B, and MHCC97L cells were cultured in Dulbecco's modified Eagle's medium (DMEM) supplemented with 10% fetal bovine serum (FBS) and antibiotics at 37°C and 5% CO_2_.

### Cell migration and invasion assays

For the migration assay, 5 × 10^4^ cells were placed in the upper chamber of each transwell insert (BD Biosciences, Franklin Lakes, NJ, USA) with a non-coated membrane. For the invasion assay, 1 × 10^5^ cells were placed in the upper chamber of each Matrigel-coated insert (BD Biosciences, Billerica, MA, USA). After incubation at 37°C for several hours, cells remaining in the upper chambers of each insert were removed with cotton swabs, and cells adhering to the lower membrane were stained with 0.1% crystal violet in 20% methanol. Cells that had migrated or invaded into the lower membrane were imaged and counted using an IX71 inverted microscope (Olympus, Tokyo, Japan).

### Vector construction

The human pri-miR-135b sequence was amplified from normal human genomic DNA and cloned into the *Bam*HI and *Eco*RI sites of the lentivirus expression vector pWPXL to generate pWPXL-miR-135b. The *RECK, EVI5, and HSF1* expression vectors were constructed by inserting the respective open reading frame sequences into the pWPXL vector to generate pWPXL-RECK, pWPXL-EVI5, and pWPXL-HSF1, respectively. The 3′ untranslated region (UTR) of *RECK and EVI5* was amplified from the genomic DNA of normal liver tissues and subcloned into the region directly downstream of a cytomegalovirus (CMV) promoter-driven firefly luciferase cassette in the pCDNA3.0 vector. Primer sequences are shown in Supplementary Table S3. All constructs were verified by sequencing.

### Lentivirus production and infection

HEK-293T cells were transfected with pWPXL-miR-135b, pWPXL-RECK, pWPXL-EVI5, or pWPXL-HSF1, along with the packaging and envelope plasmids psPAX2 and pMD2.G, respectively (gifts from Dr. Didier Trono) using Lipofectamine 2000 (Invitrogen) according to the manufacturer's instructions. Virus particles were harvested 48 h after transfection. SMMC-7721 and Huh-7 cells were infected with recombinant lentivirus-transducing units using 6 μg/ml polybrene (Sigma, St. Louis, MO, USA).

### Oligonucleotide transfection

MiR-135b mimics were synthesized by Genepharma (Shanghai, China), and the miR-135b inhibitor was synthesized by Ribobio (Guangzhou, China). Small interfering (si)RNA duplexes were designed and synthesized by Genepharma. The sequences used are shown in Supplementary Table 3. Cells were transfected using Lipofectamine 2000 and collected 48 h later for migration and luciferase reporter assays and western blotting.

### *In vivo* metastasis assays

For *in vivo* metastasis assays, 2 × 10^6^ SMMC-7721 cells infected with pWPXL -miR-135b or empty vector were resuspended in 40 μl of serum-free DMEM with Matrigel (1:1) for each male BALB/c-nu/nu mouse (n = 8 per group). Under anesthesia, each animal was orthotopically inoculated in the left hepatic lobe using a microsyringe through an 8-mm transverse incision in the upper abdomen. After 8 weeks, mice were sacrificed and their livers and lungs were dissected, fixed with phosphate-buffered neutral formalin, and prepared for histological examination. Mice were handled and housed according to protocols approved by the Shanghai Medical Experimental Animal Care Commission.

### Luciferase reporter assay

HEK-293T cells were seeded in 96-well plates at a density of 5 × 10^3^ cells per well 24 h before transfection. Cells were transfected with a mixture of 50 ng firefly luciferase reporter, 5 ng pRL-CMV Renilla luciferase reporter, and 5 pmol miRNA mimic. Cells were lysed 48 h later and firefly and Renilla luciferase activities were measured using the dual-luciferase reporter assay (Promega, Madison, WI, USA).

### DNA and RNA extraction, and quantitative real-time polymerase chain reaction

Genomic DNA was isolated using the QIAamp DNA Mini Kit (Qiagen, Valencia, CA, USA), and total RNA was extracted using TRIzol reagent (Invitrogen, Carlsbad, CA, USA). For mRNA detection, complementary DNA was synthesized using the PrimeScript RT reagent kit (Takara Bio Inc., Dalian, China) and RT-PCR was performed using SYBR Premix Ex Taq (Takara Bio Inc.). To detect mature miR-135b, RNA was reverse-transcribed with reverse transcription primers (Applied Biosystems, Foster City, CA, USA), and quantitative PCR was performed to quantitate the expression level of mature miR-135b with a TaqMan miRNA assay (Applied Biosystems) using small nuclear U6B RNA as an internal standard.

### Cell proliferation and colony formation assays

Cell proliferation assay was performed with Cell Counting Kit-8 (Dojindo Laboratories, Kumamoto, Japan). 1,000 cells were plated into 96-well plates. Ten microliters of CCK-8 solution were added into each well every day. After 2 hours of incubation at 37°C,the absorbance at 450nM was measured.

For the colony formation assays, cells were trypsinized and 1000 cells were plated onto 6-well plates and incubated for 14 days. Colonies were dyed with dyeing solution containing 0.1% crystal violet and 20% methanol and counted using inverted microscope.

### Gelatin zymography

To evaluate the activities of matrix metalloproteinases (MMP)2 and 9, cells were seeded in 6-well plates and incubated for 24 h. The supernatant was collected and mixed with sample buffer, and resolved by 8% SDS-PAGE with 1 mg/ml gelatin. Gels were incubated for 1 h at room temperature in renaturing buffer and then in developing buffer overnight at 37°C. Gels were stained with Coomassie blue for 30 min, followed by incubation in destaining solution (50% methanol and 10% acetic acid).

### Cell-matrix adhesion assay

Matrigel (1 mg/ml) was used to coat 96-well plates overnight, which were then blocked with 1% bovine serum albumin (BSA). SMMC-7721 and Huh-7 cells were trypsinized and resuspended in DMEM and then seeded at 2 × 10^4^ cells/well in the coated plates, with BSA-coated wells and cells incubated in complete culture medium for 6 h serving as negative and positive controls, respectively. After incubation at 37°C for 1 h, unattached cells were washed away and attached cells were fixed with 70% ethanol and stained with 0.1% crystal violet in 20% ethanol. The crystal violet was dissolved in 10% acetic acid, and absorbance was measured at 595 nm. Experiments were repeated at least three times.

### Fluorescence labeling and imaging

SMMC-7721 and Huh-7 cells were seeded on glass slides and cultured for 24 h, then fixed with 4% paraformaldehyde and blocked in phosphate-buffered saline containing 10% FBS for 1 h at room temperature. F-actin was detected with phalloidin-Alexa Fluor 594 (Life Technologies, Carlsbad, CA, USA) and nuclei were visualized by counterstaining with 4′,6-diamidino-2-phenylindole (Sigma). The actin cytoskeleton was visualized and imaged under an Olympus FV1000 confocal microscope.

### Western blotting

Proteins were separated by sodium dodecyl sulfate-polyacrylamide gel electrophoresis (SDS-PAGE) and transferred to a nitrocellulose membrane (Bio-Rad, Hercules, CA, USA), which was blocked and probed with primary antibodies against RECK or HSF1 (both from Cell Signaling Technology, Danvers, MA, USA) or EVI5 (Thermo Fisher Scientific, Waltham, MA, USA). Immune complexes were detected by enhanced chemiluminescence (Pierce, Rockford, IL, USA).

### Chromatin immunoprecipitation

A chromatin immunoprecipitation assay kit (Thermo Fisher Scientific) was used according to the manufacturer's protocol. Briefly, SMMC-7721 cells were treated with 1% formaldehyde for 10 min at room temperature followed by glycine. Lysates were then treated with micrococcal nuclease, and protein-DNA complexes were precipitated with an anti-HSF1 antibody or control IgG. After overnight incubation at 4°C, complexes were purified and crosslinks were reversed at 65°C. Purified DNA was amplified by PCR using the primers listed in Supplementary Table 3.

### Electrophoretic mobility shift assay (EMSA)

Nuclear extracts were prepared with the NE-PER nuclear extraction reagent(Thermo Fisher Scientific, Waltham, MA, USA). Electrophoretic mobility shift assay (EMSA) was carried out according to the protocol accompanying EMSA/Gel-Shift kit (Beyotime Biotechnology). Biotin end-labeled DNA duplex(Supplementary Table S3) containing a putative binding site for HSF1 was incubated with the nuclear extracts. The competition reactions were performed by adding 200-fold excess unlabeled consensus oligonucleotide to the reaction mixture. The reactions were transferred to a nylon membrane. The biotin-labeled DNA was detected with LightShift chemiluminescent electrophoretic mobility shift assay kit (Thermo Fisher Scientific). For supershift assays, the nuclear extract (3μg) was incubated at room temperature for 20 min with biotin-labeled probe and antibody against HSF1 (Cell Signaling Technology, Danvers, MA, USA). Normal immunoglobulin G (IgG) was as a control. DNA/nuclear protein complexes were separated by electrophoresis on a native 6% acrylamide gel.

### Statistical analysis

Statistical analyses were performed using GraphPad Prism 5(GraphPad Software, Inc., San Diego, CA, USA) and SPSS v.20.0(SPSS Inc., Chicago, IL, USA). The results were presented as mean ± SEM. The data were subjected to Student's *t*-test (P < 0.05 was considered statistically significant) unless otherwise specified (paired *t*-test, χ^2^ test).

## SUPPLEMENTARY MATERIAL, FIGURES, TABLES


