# Supplementary material for: MicroRNA-135b, a HSF1 target, promotes tumor invasion and metastasis by regulating RECK and EVI5 in hepatocellular carcinoma
Source: Oncotarget. 2014 Dec 11;6(4):2421–33. doi: 10.18632/oncotarget.2965 (PMC4385861; doi:10.18632/oncotarget.2965)
Supplement: Supplementary file 1 [file oncotarget-06-2421-s001.pdf]

MicroRNA-135b, a HSF1 target, promotes tumor invasion and metastasis by regulating RECK and EVI5 in hepatocellular carcinoma

Supplementary Material

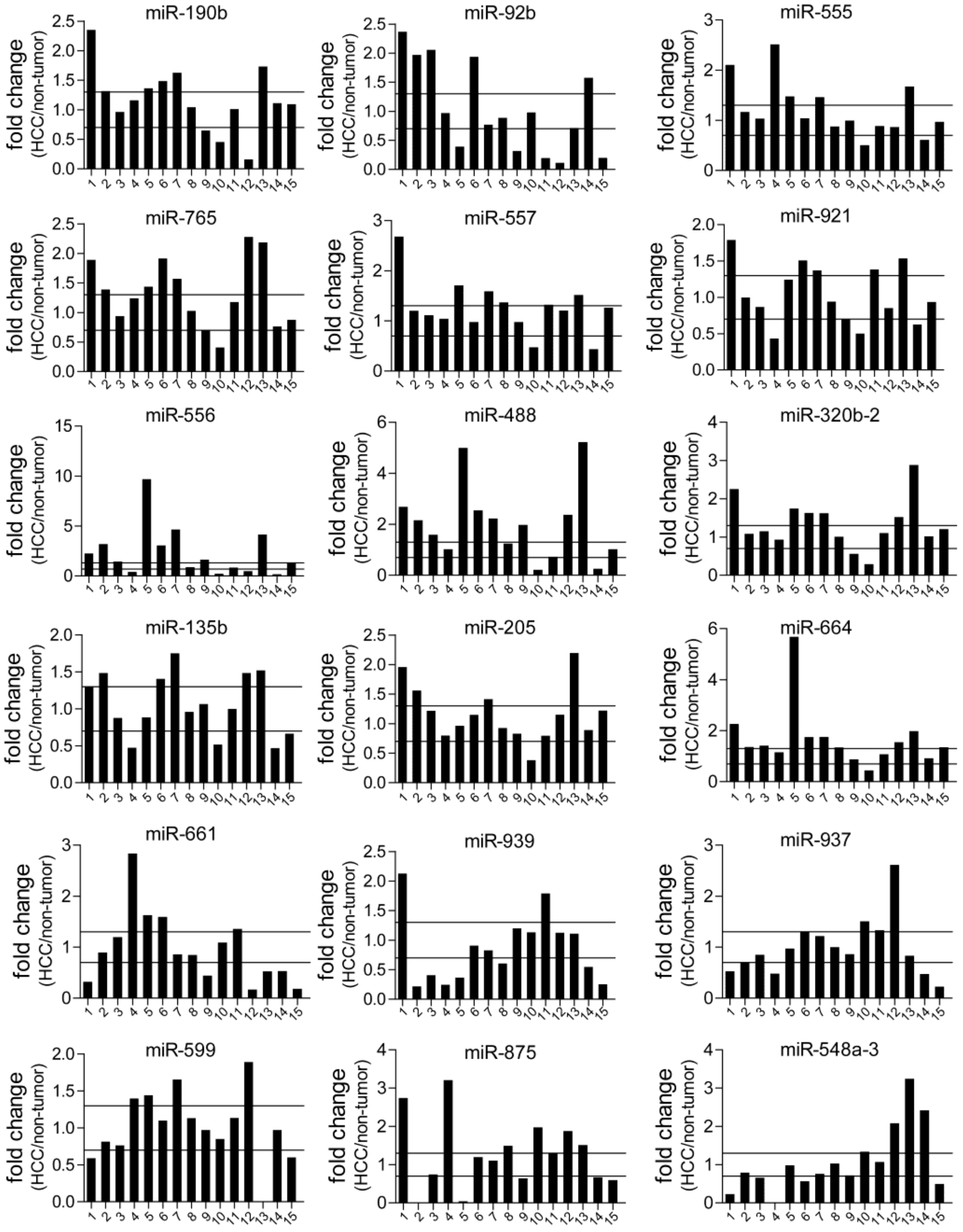

Supplementary Figure S1: Genomic DNA content of aberrantly expressed miRNAs in hepatocellular carcinoma (HCC). Analysis of copy number for 18 miRNAs aberrantly expressed in HCC and adjacent noncancerous liver tissue samples (n = 15) by real-time PCR; expression levels were normalized to β-actin.

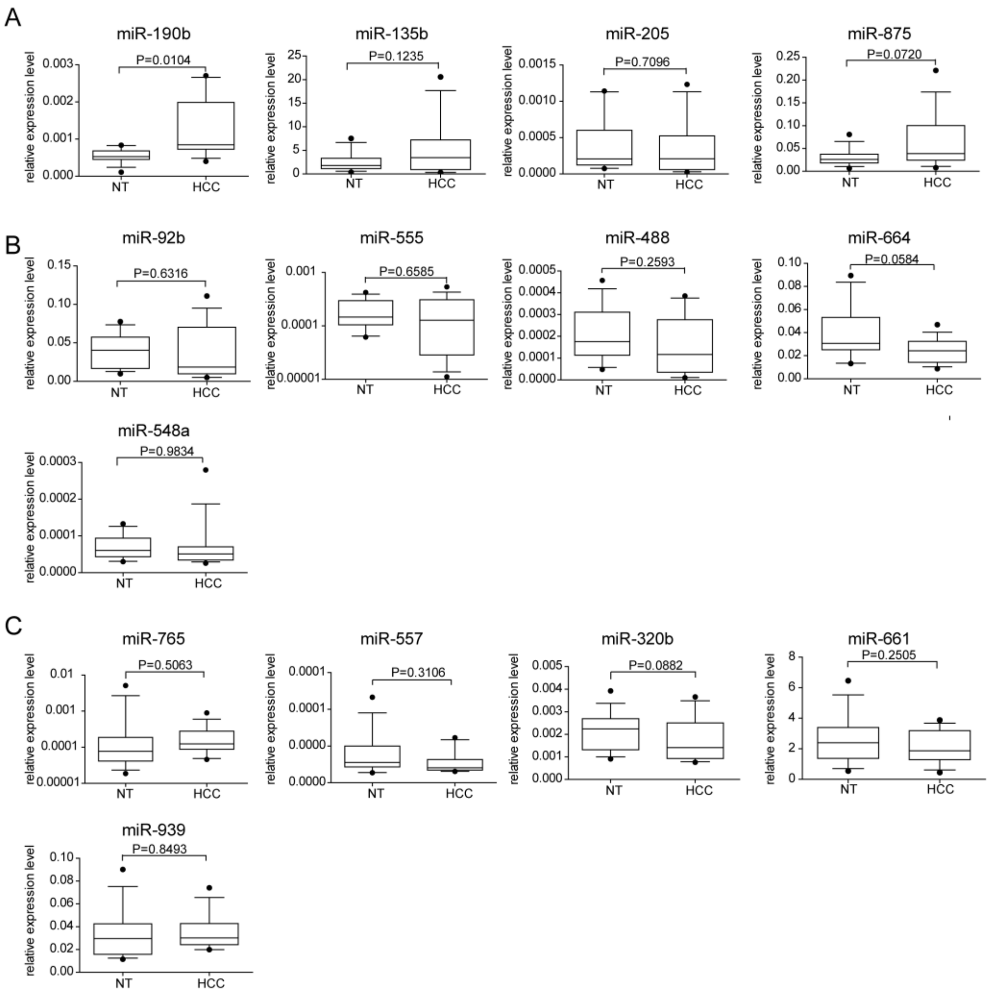

Supplementary Figure S2: Expression of miRNAs aberrantly expressed in hepatocellular carcinoma (HCC).

Transcript expression levels of 18 miRNAs aberrantly expressed in HCC and corresponding noncancerous liver tissue samples (NT) (n = 14); no signal was detected for four of the miRNAs. Lower and upper boxes define the 10th and 90th percentiles, respectively, with the median value indicated by a line. (**A**) miRNAs with upregulated expression (fold change of HCC vs NT >2, frequency > 35%). (B) miRNAs with downregulated expression (fold change of HCC vs NT < 0.5, frequency > 35%). (C) miRNAs with unchanged expression (fold change of HCC vs NT >2 or fold change of HCC vs NT < 0.5, frequency < 35%).

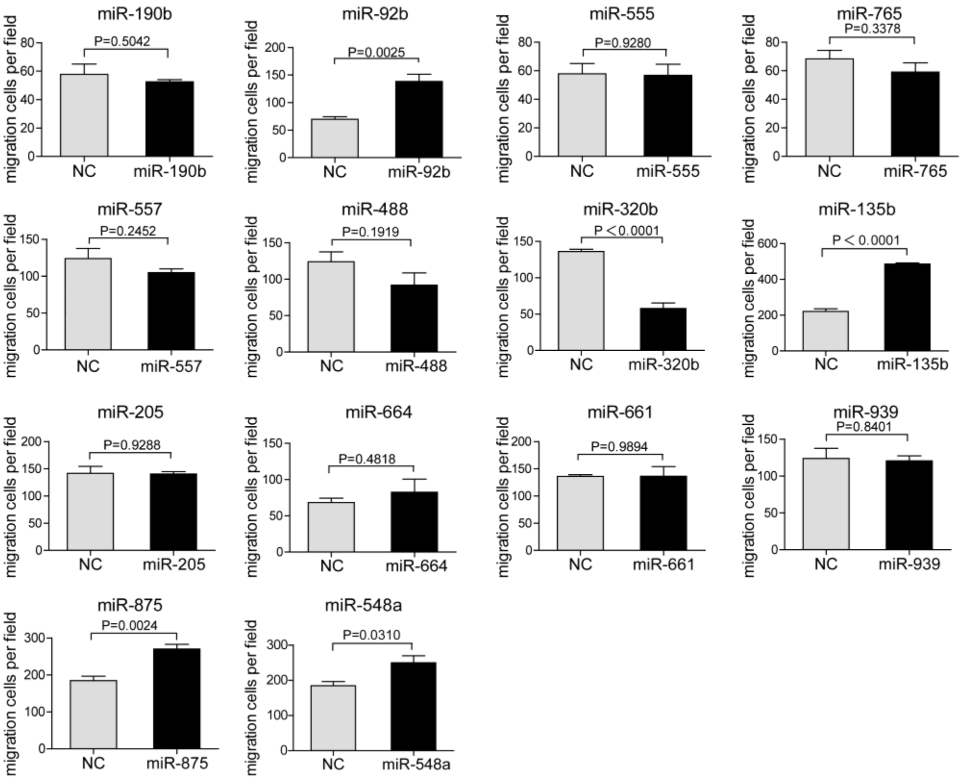

Supplementary Figure S3: Cell migration following transfection with miRNAs aberrantly expressed in hepatocellular carcinoma (HCC).

Motility of Huh-7 cells transfected with miRNA mimics or negative control (NC) was evaluated by the transwell migration assay. Data represent the mean ± SEM. $P < 0.05$ (Student's *t* test) was considered statistically significant.

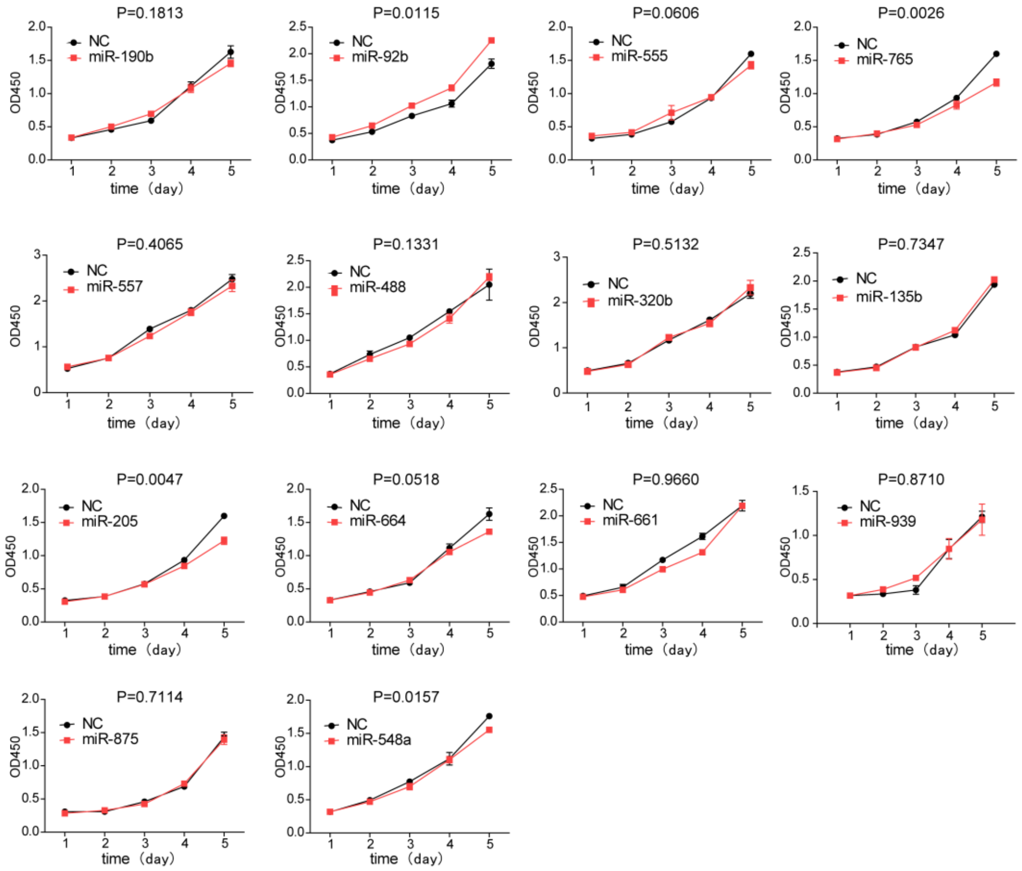

Supplementary Figure S4: Cell proliferation following transfection with miRNAs aberrantly expressed in hepatocellular carcinoma (HCC).

Huh-7 cell proliferation was assessed after transfection with miRNA mimic or the negative control (NC). Data represent mean ± SEM of three independent experiments. $P < 0.05$ (Student's t test) was considered statistically significant.

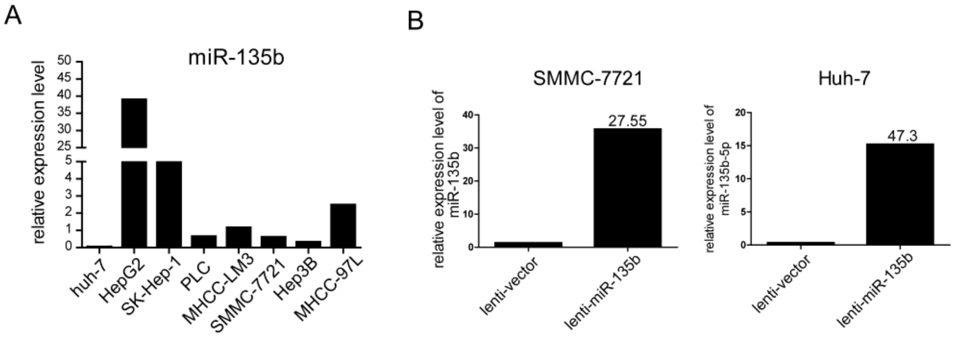

Supplementary Figure S5: MiR-135b expression in various hepatocellular carcinoma (HCC) cell lines and establishment of stable cell lines.

(A) MiR-135b transcript level was detected by TaqMan real-time PCR relative to the internal standard small nuclear U6B RNA (RNU6B). (B) MiR-135b transcript levels relative to RNU6B in stable cell lines expressing miR-135b or control lentivirus.

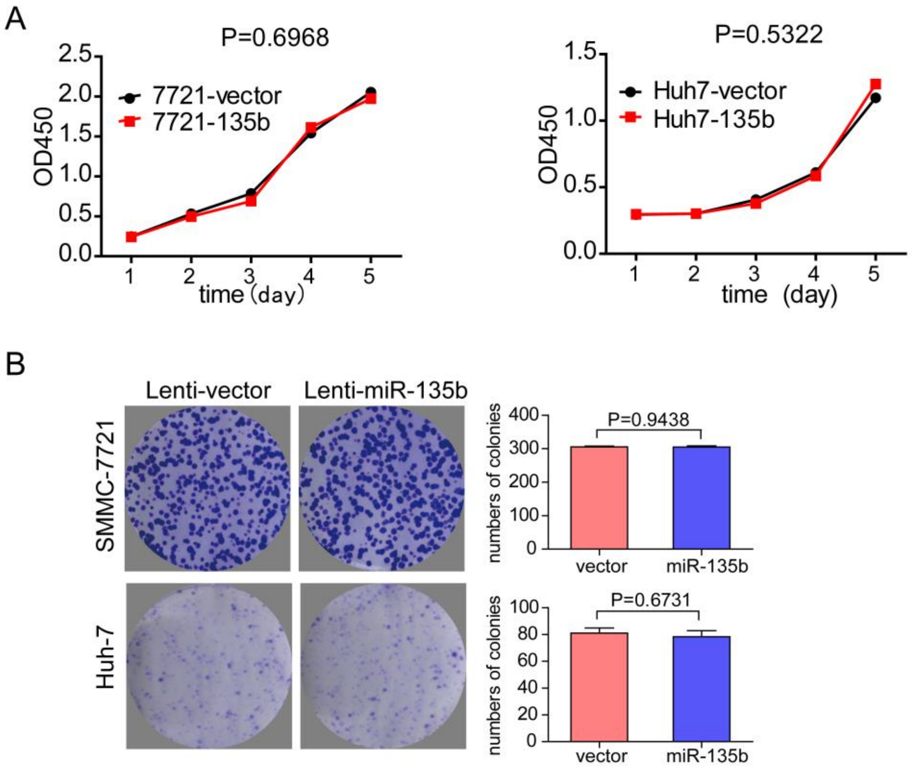

Supplementary Figure S6: MiR-135b overexpression has no effect on hepatocellular carcinoma (HCC) cell growth in vitro.

(A,B) Cell proliferation in SMMC-7721 and Huh-7 cells transduced with miR-135b or control lentivirus was assessed with the Cell Counting Kit 8(A) and colony formation assay(B). Data represent the mean ± SEM of triplicate samples.

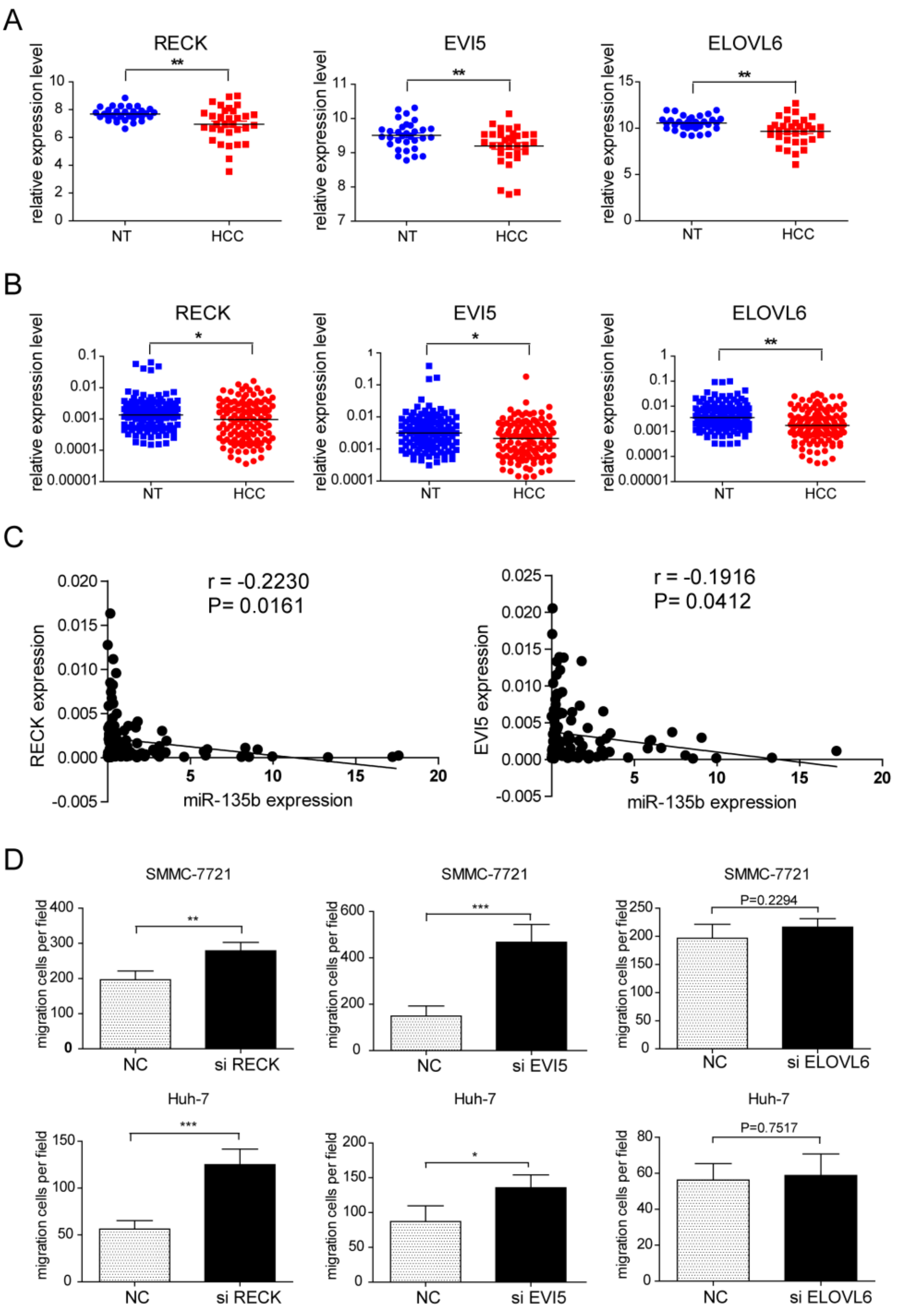

Supplementary Figure S7: Identification of miR-135b target genes.

(A) Transcript expression level of candidate target genes in The Cancer Genome Atlas (TCGA; http://tcga.cancer.gov) and three genes—reversion-inducing-cysteine-rich protein with kazal motifs (RECK), ecotropic viral integration site5 (EVI5) and fatty acid elongase (ELOVL)6,—downregulated in hepatocellular carcinoma (HCC; n = 32). *P < 0.05; **P < 0.01; ***P < 0.001 (paired t-test). (B) Relative expression

levels of RECK, EVI5 and ELOVL6 in 120 paired HCC and adjacent noncancerous liver tissue samples (NT), as assessed by real-time PCR. (C) Correlation between expression level of RECK or EVI5 and that of mature miR-135b in 120 HCC tissues. Linear regression coefficient and statistical significance is indicated. (D) Transwell migration assay of SMMC-7721 and Huh-7 cells transfected with small interfering (si)RNA against RECK, EVI5, or ELOVL6 or with a negative control (NC).

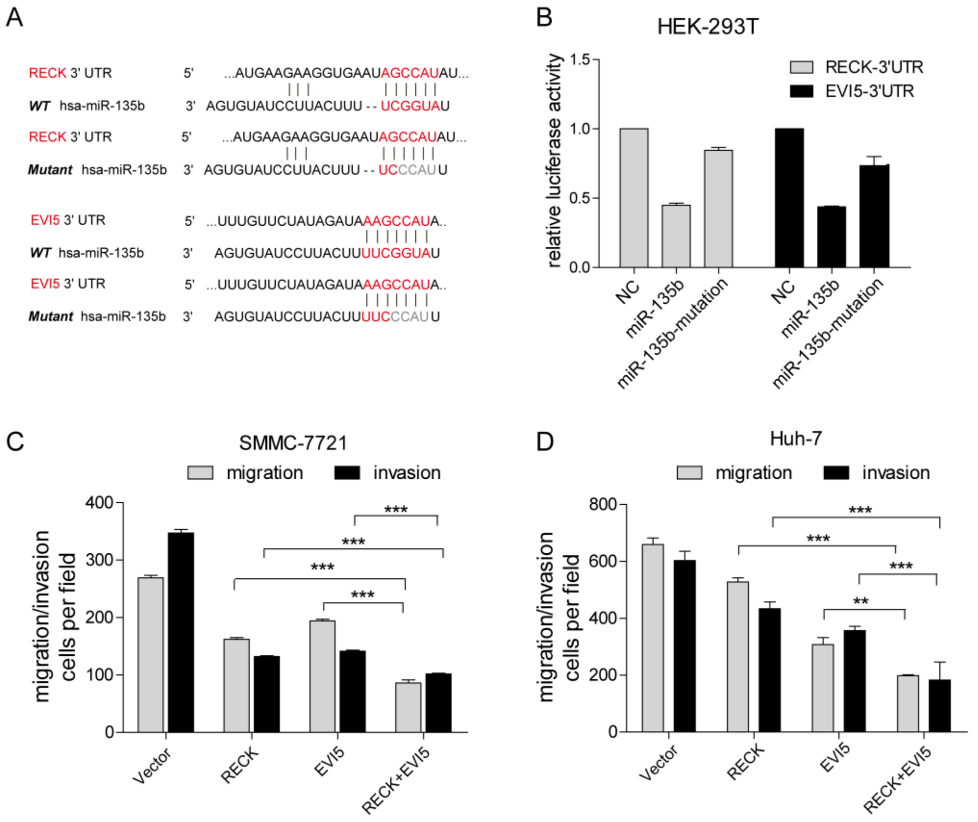

Supplementary Figure S8: **RECK and EVI5 are direct targets of miR-135b and they reduce the migration and invasion ability of HCC cells.**

(A) A compensatory mutation was synthesized in miR-135b. Mutant binding sequences were highlighted in gray. (B) HEK-293T cell was co-transfected with luciferase reporter of RECK or EVI5's 3' UTR and wild-type or mutant miR-135b mimic. Firefly luciferase activity was normalized to Renilla luciferase activity. (C,D) Transwell migration and invasion assays of SMMC-7721 and Huh-7 cells after RECK or EVI5 or both the two target genes overexpression. *P < 0.05; **P < 0.01; ***P < 0.001. Student's t test.

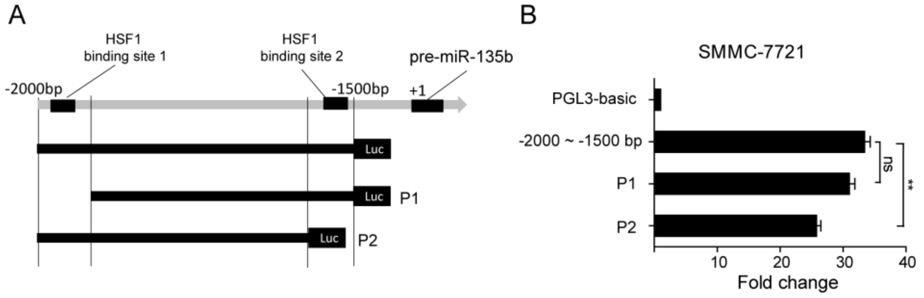

Supplementary Figure S9: The transcription factor HSF1 regulates miR-135b. (A) Schematic for firefly luciferase reporter constructs. P1 and P2 fragments were cloned to the pGL3-basic vector. P1 represented HSF1 binding site 1 deletion . P2 represented HSF1 binding site 2 deletion. (B) Luciferase activity in SMMC-7721 after HSF1 binding site 1 or 2 deletion. Firefly luciferase activity was normalized to Renilla luciferase activity.

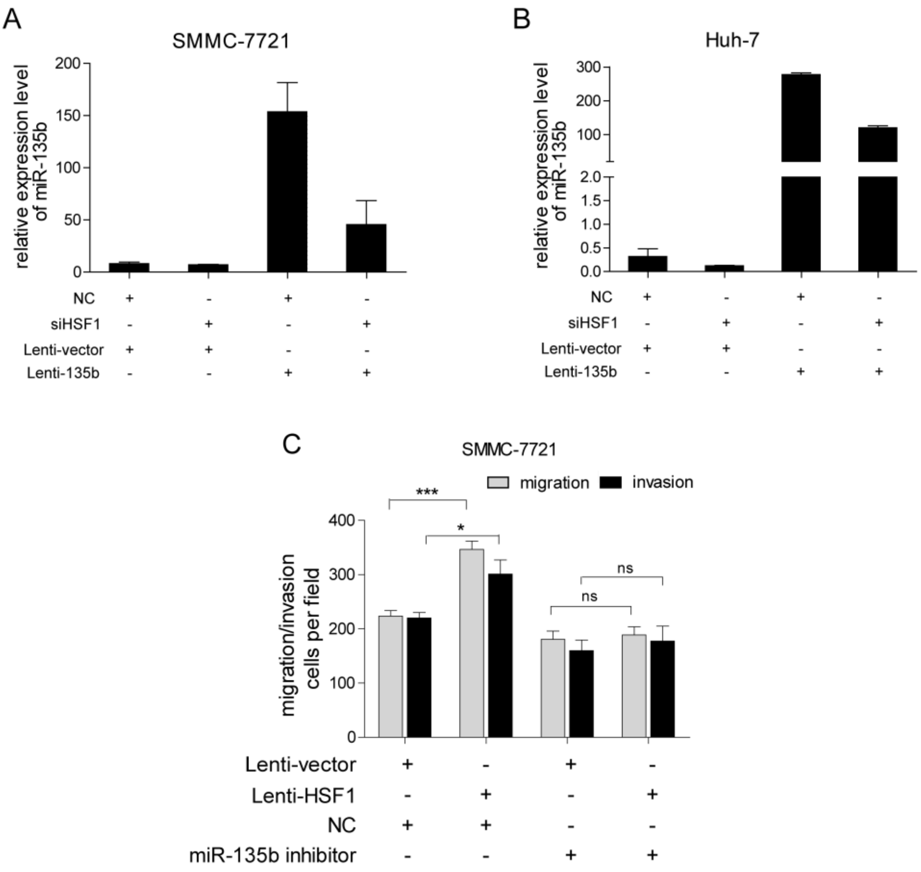

Supplementary Figure S10: HSF1 promotes hepatocellular carcinoma (HCC) cell migration and invasion via miR-135b.

(A,B) Expression of miR-135b was assessed by real-time PCR in SMMC-7721(A) and Huh-7(B) after restoration of miR-135b following HSF1 knockdown. U6B RNA (RNU6B) as an internal control. (C) SMMC-7721 cells were transfected with miR-135b inhibitor or a negative control (NC) after transduced with HSF1 or control lentivirus.*P < 0.05; **P < 0.01; ***P < 0.001. Statistical analysis was performed with Student’s t test.

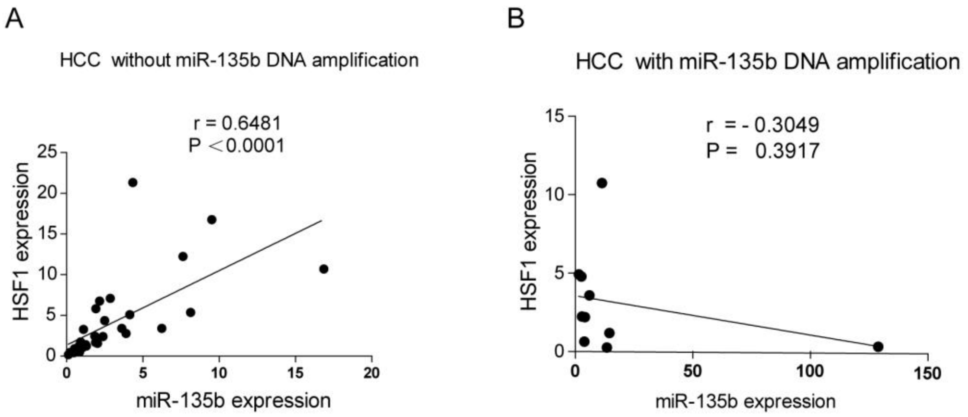

Supplementary Figure S11: Correlation between miR-135b and HSF1 expression in HCC sample with or without miR-135b DNA amplification.

Genomic region alternation of miR-135b, the levels of miR-135b and HSF1 expression were analyzed in a cohort of 42 pairs of HCC samples. miR-135b DNA amplification occurred in 10 pairs of HCCs. (A) HSF1 was positively correlated with the expression of miR-135b in the HCC sample without miR-135b DNA amplification (n=32).(B) HSF1 had no correlation with miR-135b expression in the HCC sample with miR-135b DNA amplification(n=10).

Supplementary Tables

Supplementary Table S1: 18 microRNAs Screened from SNP 6.0 arrays

miRNA name	CNV locus	Amplification A, Deletion D, (frequency, %)	Mature miRNA alteration (frequency, %)	Migration (promote P, inhibit I)	Proliferation (promote P, inhibit I)
hsa-mir-190b	chr1:152031834-153219437	A(40.00)	up(42.86)	ns	ns
hsa-mir-92b	chr1:153222188-154965896	D(33.33)	down(42.86)	P（**）	P（*）
hsa-mir-555	chr1:153222188-154965896	A(33.33)	down (35.71)	ns	ns
hsa-mir-765	chr1:154965896-155306435	A(46.67)	up(28.57)	ns	I（**）
hsa-mir-557	chr1:166182288-166671151	A(42.86)	down(28.57)	ns	ns
hsa-mir-921	chr1:162616201-165028985	A(33.33)	no signal	—	—
hsa-mir-556	chr1:160204382-160870855	A(53.33)	no signal	—	—
hsa-mir-488	chr1:172990641-177176632	A(60.00)	down(57.14)	ns	ns
hsa-mir-320b-2	chr1:222055484-224353955	A(42.86)	down(21.43)	I（***）	ns
hsa-mir-135b	chr1:203295912-208449143	A(33.33)	up(57.14)	P（***）	ns
hsa-mir-205	chr1:203295912-208449143	A(28.57)	up(35.71)	ns	I（**）
hsa-mir-664	chr1:216336382-218993297	A(66.67)	down(50)	ns	ns
hsa-mir-661	chr8：143851512-146268960	D(42.86)	down(14.29)	ns	ns
hsa-mir-939	chr8：143851512-146268960	D(46.67)	up(21.43)	ns	ns
hsa-mir-937	chr8：143851512-146268960	D(33.33)	no signal	—	—
hsa-mir-599	chr8：100454540-101472365	A(28.57)	no signal	—	—
hsa-mir-875	chr8：100454540-101472365	A(42.90)	up(50.00)	P（**）	ns
hsa-mir-548a-3	chr8：105107293-105839798	D(28.57)	down(35.71)	P（*）	I（*）

*** P< 0.001, ** P=0.001 to 0.01, * P=0.01 to 0.05, ns： Not significant P>0.05

Supplementary Table S2: Correlation of the Clinicopathological Features With Tumor miR-135b Expression in HCC

	n	miR-135b expression Up-regulation (＞Median)	Down-regulation (＜Median)	χ^2	P Value
**Gender**					
Male	99	49	50	0.667	0.414
Female	13	8	5		
**Age (years)**					
≤55	78	42	36	0.267	0.605
＞55	33	16	17		
**Hepatitis**					
Positive	67	31	36	0.672	0.412
Negative	52	28	24		
**Cirrhosis**					
Positive	26	15	11	0.706	0.401
Negative	91	44	47		
**HBsAg**					
Positive	84	41	43	2.625	0.105
Negative	22	15	7		
**Anti-HBs**					
Positive	10	7	3	1.691	0.193
Negative	89	43	46		
**HBeAg**					
Positive	20	6	14	4.412	0.036 *
Negative	80	45	35		
**Anti-HBeAg**					
Positive	45	25	20	0.842	0.359
Negative	54	25	29		
**Anti-HBc**					
Positive	72	39	33	0.722	0.396
Negative	29	13	16		
**Anti-HCV**					
Positive	7	3	4	0.088	0.766
Negative	78	38	40		
**Serum AFP (ng/ml)**					
1≤20	32	17	15	0.399	0.528
2 ＞20	69	32	37		
**Tumor size (cm)**					
＞5	37	21	16	0	0.994
≤5	30	17	13		
**Tumor capsule**					
Positive	53	22	31	4.215	0.04 *

Negative	52	32	20		
**Lymphatic Invasion**					
Positive	5	3	2	0.172	0.679
Negative	99	50	49		
**Distant metastasis**					
Positive	3	2	1	0.269	0.604
Negative	101	52	49		

Note: The total numbers were not consistent in different categories because we lacked some information of the HCC samples. *, χ2 test, P<0.05

Supplementary Table S3: Primers sequences for real time PCR and constructs

	Forward primer(5'-3')	Reverse primer(5'-3')
**copy number of 18**		
**miRNA**		
has-miR-190b	GTCTGCTCTATTCTTTCTTTGC	TGTTTGACATTTAGTTGGTTCC
has-miR-92b	CTCCGCAAACTCCCGTTCTCCC	CAACACTGCACCGCGTCCCGTC
has-miR-555	GTTTTTCTACAGGTATGGCAGC	ATCAGAGTTTTATCAGAGGTTC
has-miR-765	GACAGCCCTTTTCAAGCCCT	AGTCCCTGAAGGAGGAGTGG
has-miR-557	AAGTGTTATCGGACCAAGGGGG	TCCTTTCAAAGACAAGGCCCAC
has-miR-921	CAGGGTGTTAGGATGGTGCGGG	CCAGTGATCCAGGCCCATGGAC
has-miR-556	GAACATAGAAATGACTCCAGGC	GTTGAAGGTAGTAATAAAAAAG
has-miR-488	AGGAGCAGGAGGCGTAGTAGAG	TTTGGAAACTTGTTTGAGAGTG
has-miR-320b-2	TTTGAGGGGAGGTAGGTGCT	TTTTGCCCTCTCAACCCAGC
has-miR-135	TTCCCTATGAGATTCCTGC	CAAAGCCTCCTTCTGGTG
has-miR-205	TGTGATCCTATGGGAGGGGTAGG	CCACTGAAATCTGGTTGGGTATG
has-miR-664	AAAGAGCAAGGAGAAGAGGAAGG	GGCTGGGGATAAATGAATAGAAT
has-miR-661	TCAACGTGCATTCTCCTGGG	CCCCACAGCACAGCCTC
has-miR-939	CCTCTGCTCCCCAGTGTCTGACC	CTCCCACACGACCGACTTTTTGC
has-miR-937	GAGCTGGGTGTGGGGGCGTATAG	GCAGAGAGTCAGAGCGCGGATGG
has-miR-599	CATAAGCTTCGTGACTTCTATTC	GGTTTGATAAACTGACACAACAG
has-miR-875	CATCGAACTGAACAATAA	AATAGAAGTCACGAAGCT
has-miR-548a-3	GAATATACTCCCCAAAGAACTC	TGAAAGTAATGGTAAAACTCGC
β-actin Genomic qPCR	TGACTTAGTTGCGTTACACCCTT	CACCTTCACCGTTCCAGTTTT
**3'UTR of target genes**		
RECK-outside	CTCTGCCCTCATCATTTC	CCTCAGCCTCCCAAGTAG
RECK-inside	CCGGAATTCCGG CTTCTTGCCACTCCCTCC	CGCGGATCCGCG TCCATGACTTCTTATCCC
RECK-Mntant	AAGAAGGTGAATGCAGATATTTGTAAAATGA	TCATTTTACAAATATCTGCATTCACCTTCTT
EVI5-outside	TTTTATGTGCATATGATGCTTC	AATCTTGGCTCTGCTACTTACT
EVI5-inside	CGGGGTACCCCGCACCTAACATTGAACATC	CGCGGATCCGCGGCAAATAGCTACAAAAAC
EVI5-Mutant	GTTCTATAGATAGGATCATAAGAATCCTTTCC	GGAAAGGATTCTTATGATCCTATCTATAGAAC
**siRNA of target genes**		
RECK siRNA-1	AAGUGAAUCCCGACUAAAATT	UUUUAGUCGGGAUUCACUUTT
RECK siRNA-2	CUCGGUUUGUUGCAGUUAUTT	AUAACUGCAACAAACCGAGTT
RECK siRNA-3	CCUCAGGCCAAGUACUUUATT	UAAAGUACUUGGCCUGAGGTT
EVI5 siRNA-1	GGACUCCUUACUCAAUUAATT	UUAAUUGAGUAAGGAGUCCTT
EVI5 siRNA-2	GAGUCUCAGUGUGCAUUAATT	UUAAUGCACACUGAGACUCTT
EVI5 siRNA-3	GGCUUACUCUUUAGUAGAUTT	AUCUACUAAAGAGUAAGCCTT
ELOVL6 siRNA-1	GCUCUGUAUGCUGCCUUUATT	UAAAGGCAGCAUACAGAGCTT
ELOVL6 siRNA-2	GCCAUUAGUGCUCUGGUCUTT	AGACCAGAGCACUAAUGGCTT
ELOVL6 siRNA-3	CUGGUGCUUAUAUGGUGUATT	UACACCAUAUAAGCACCAGTT
**ORF**		
RECK(ORF)	CGACGCGTCG AAGCTGGGTCCGAGCATCC	TCCCCCGGGGGA GTGGAGGAGCATTCTGCACT

EVI5(ORF)	CGCGGATCCGCG ATGGTTACCAACAAAATG	CCGGAATTCCGG TCAGACAGTGGTTGAATA
HSF1(ORF)	CGACGCGTCGCTCCGCCTATTCCCTCC	CCGGAATTCCGGGCCCGTTTGTCCTACTG
**promoter regions**		
-3000 ~ +1 bp	CGGGGTACCCCGATGAGGGACAATGGGAGCC	TCCCCCGGGGGAAAAGCCATAGGCCACAGCAG
-1500 ~ + 1 bp	CGGGGTACCCCGGAGTGGAGGAGTGAGTGGC	TCCCCCGGGGGAAAAGCCATAGGCCACAGCAG
-3000 ~ -1500 bp	CGGGGTACCCCGATGAGGGACAATGGGAGC	TCCCCCGGGGGAAGCCACTCACTCCTCCAC
-3000 ~ - 2500 bp	CGGGGTACCCCGATGAGGGACAATGGGAGCC	TCCCCCGGGGGATGAAAGGCCACTGCTGGG
-2500 ~ -2000 bp	CGGGGTACCCCGAGTGGCCTTTCACTGCCC	TCCCCCGGGGGAGGCAGGAGACCTGAGACG
-2000 ~ -1500 bp	CGGGGTACCCCGCGTCTCAGGTCTCCTGCC	TCCCCCGGGGGAAGCCACTCACTCCTCCAC
**ChIP**		
Binding site 1	TGCCAAAAATTAAACTATA	CTCACTCCCTCTGAACCAG
Binding site 2	GGGTACAGCTCAGCCCTG	CACTCACTCCTCCACTCA
E**MSA**		
Binding site 2	CCTCGCTGGGGCGTGGAGCTTCTCGGGGTGAGTGGAGGAGT	ACTCCTCCACTCACCCCGAGAAGCTCCACGCCCCAGCGAGG
